# Mcl-1 expression is a predictive marker of response to gemcitabine plus nab-paclitaxel for metastatic pancreatic cancer

**DOI:** 10.1038/s41598-024-73020-8

**Published:** 2024-09-20

**Authors:** Makiko Urabe, Kenji Ikezawa, Yusuke Seiki, Ko Watsuji, Yasuharu Kawamoto, Takeru Hirao, Yugo Kai, Ryoji Takada, Takuo Yamai, Kaori Mukai, Tasuku Nakabori, Hiroyuki Uehara, Shigenori Nagata, Kazuyoshi Ohkawa

**Affiliations:** 1 Department of Hepatobiliary and Pancreatic Oncology, Osaka International Cancer Institute, 3-1-69 Otemae, Chuo-ku, Osaka, 541-8567 Japan; 2 Department of Diagnostic Pathology and Cytology, Osaka International Cancer Institute, Osaka, Japan

**Keywords:** Anti-apoptotic protein, Chemotherapy, Endoscopic ultrasound-guided fine-needle aspiration, Liver tumor biopsy, Predictive marker, Cancer, Gastroenterology, Molecular medicine, Oncology

## Abstract

Antiapoptotic protein, including Mcl-1, expression is frequently observed in pancreatic cancer. Gemcitabine plus nabpaclitaxel (GnP) is the standard chemotherapy for metastatic pancreatic cancer (MPC); however, predictive markers for its efficacy remain unestablished. This study evaluated the association between GnP’s therapeutic effects and Mcl-1 expression in tissue samples obtained using endoscopic ultrasound-guided fine-needle aspiration (EUS-FNA) for pancreatic tumor or percutaneous ultrasound-guided biopsy for metastatic liver tumor. We retrospectively reviewed 38 patients with histologically diagnosed MPC who received GnP as the first-line chemotherapy at our institute between December 2014 and July 2018. Post-immunohistochemistry analysis for Mcl-1 expression detection, patients were divided to into two groups based on the cell proportion showing Mcl-1 immunoreactivity: positive (> 20%; 23 [60.5%] patients) and negative (≤ 20%; 15 [39.5%] patients) groups. Clinical characteristics did not differ between the two groups. The Mcl-1 positive group showed a significantly higher disease control rate (95.7% vs. 73.3%; *P* = 0.046), longer progressionfree survival (PFS) (7.2 months vs. 4.9 months; *P* = 0.018) and longer overall survival (OS) (14.9 months vs. 9.2 months; *P* = 0.008) than the Mcl-1 negative group. Multivariate analysis showed that Mcl-1 expression was an independent predictive marker for PFS and OS. Mcl-1 expression could be a predictive marker for favorable response to GnP.

## Introduction

Metastatic pancreatic cancer (MPC) accounts for approximately half of all cases of pancreatic cancer (PC) and has a 5-year survival rate of 3%^1^. Systemic chemotherapy, such as gemcitabine plus nab-paclitaxel (GnP) or modified FOLFIRINOX, is recommended as the first-line chemotherapeutic regimen for advanced PC^2–4^. GnP can be administered as systemic chemotherapy for patients with MPC in the > 75 years age group, which is the age group of a substantial proportion of patients with MPC^4–7^. Although GnP is an important treatment option for advanced PC, the available data regarding predictive markers for its therapeutic efficacy are limited^8,9^.

The apoptosis machinery, which causes programmed cell death, is regulated by the balance between pro-apoptotic and anti-apoptotic members of the Bcl-2 family^10,11^. Mcl-1 is an anti-apoptotic protein that binds to and inhibits the apoptosis-inducing proteins Bak and Bax, thereby exerting anti-apoptotic effects^12,13^. Avoidance of apoptosis is one of the hallmarks of cancer and contributes to both tumor progression and resistance to treatment^14^. Overexpression of Mcl-1 is frequently observed in human carcinomas^15,16^. Several important oncogenic pathways and transcriptional or post-transcriptional mechanisms induce increased expression of Mcl-1^17–19^. Expression of anti-apoptotic proteins, such as Bcl-xL and Mcl-1, is elevated in PC [20]. PC with a high Bcl-xL expression is reported to have a poorer prognosis than that with a low Bcl-xL expression [21,22]. However, the association between Mcl-1 expression and the therapeutic efficacy of chemotherapy for PC has not been reported.

Endoscopic ultrasound-guided fine-needle aspiration (EUS-FNA) for pancreatic tumor or percutaneous ultrasound-guided biopsy for metastatic liver tumor are pathological diagnostic modalities that are widely used to facilitate the differential diagnosis of pancreatic solid tumors^20,21^. The aim of this study was to examine the association between the therapeutic effects of GnP and the expression of Mcl-1 in tissue samples obtained using EUS-FNA or percutaneous ultrasound-guided liver tumor biopsy.

## Methods

### Study design and ethical considerations

This retrospective single-center study was approved by the Institutional Regimen Committee and Institutional Review Board of Osaka International Cancer Institute (19009-4) and conducted in accordance with the principles of the Declaration of Helsinki. The requirement for informed consent was waived by the Institutional Review Board of Osaka International Cancer Institute due to the retrospective nature of the study.

### Patients and clinical data

Among the 505 patients with advanced PC who underwent GnP at the Osaka International Cancer Institute between December 2014 and July 2018, we reviewed the clinical data of 96 patients with MPC who were pathologically diagnosed with pancreatic ductal adenocarcinoma and underwent GnP as the first-line chemotherapy based on the results of the analysis of tissue samples obtained using EUS-FNA or percutaneous ultrasound-guided biopsy for metastatic liver tumor. The major inclusion criteria were as follows: (1) patients received at least two cycles of GnP as the first-line chemotherapy for MPC; (2) sufficient histological tissue samples diagnosed as adenocarcinoma were available; (3) there was no possibility of submitting tissue samples for genetic testing due to the death of patients or disease progression. Data on clinical variables, including age, sex, Eastern Cooperative Oncology Group performance status (PS), body mass index, pancreatic tumor location, metastatic sites, serum carcinoembryonic antigen (CEA), carbohydrate antigen 19 − 9 (CA19-9), albumin levels, neutrophil-to-lymphocyte ratio (NLR), biliary drainage, and chemotherapy were obtained from the patients’ medical records. The treatment protocol for GnP involved the administration of gemcitabine 1000 mg/m^2^ on days 1, 8, and 15 and nabpaclitaxel 125 mg/m^2^ on days 1, 8, and 15. This regimen was repeated every 4 weeks. The dosages and dosing schedules were adjusted at each physician’s discretion depending on the patient’s condition. Response to chemotherapy was assessed using computed tomography and categorized as complete response, partial response, stable disease, or progression, according to the guidelines in the Response Evaluation Criteria in Solid Tumors version 1.1. Progression-free survival (PFS) was defined as the time from the start of chemotherapy to the date of tumor progression. Overall survival (OS) was defined as the date from the start of chemotherapy to the date of death.

### Acquisition of tissue samples

The specimens analyzed in this study were obtained using EUS-FNA or percutaneous ultrasound-guided liver tumor biopsy, which was performed for pathological diagnosis of PC before the initiation of chemotherapy. All EUS procedures were performed by endoscopists at our hospital using a linear-array echoendoscope (GF-UCT260; Olympus Medical Systems, Tokyo, Japan). EUS-FNA was performed with 19, 22, or 25-gauge franseen needles. The needle types, gauges and passes used were determined by the endoscopists. The aspirated biopsy materials were placed in a Petri dish with saline and into a formalin-filled container for histological analysis. Percutaneous ultrasound-guided liver tumor biopsy was performed using a 21-gauge aspiration needle (Sonopsy-C1; Hakko) or an 18-gauge core needle (MONOPTY). Thereafter, the formalin-fixed specimens were embedded in paraffin. Two experienced pathologists examined the hematoxylin and eosin-stained specimens and classified them as pancreatic ductal adenocarcinoma based on their findings.

### Immunohistochemical analysis of the specimens

The formalin-fixed paraffin-embedded blocks of PC were cut into 3–5 μm slices, and the sections were subjected to immunohistochemical analyses to evaluate the immunoreactivity of the samples to Mcl-1. A monoclonal rabbit anti-Mcl-1 antibody (1:500, #39224; Cell Signaling Technology, Danvers, MA) was used for immunohistochemical staining. Antibody binding was visualized using the autoimmunostaining system VENTANA BenchMark ULTRA (Roche Diagnostics, Wetzlar, Switzerland) according to the manufacturer’s instructions.

**Fig. 1:**
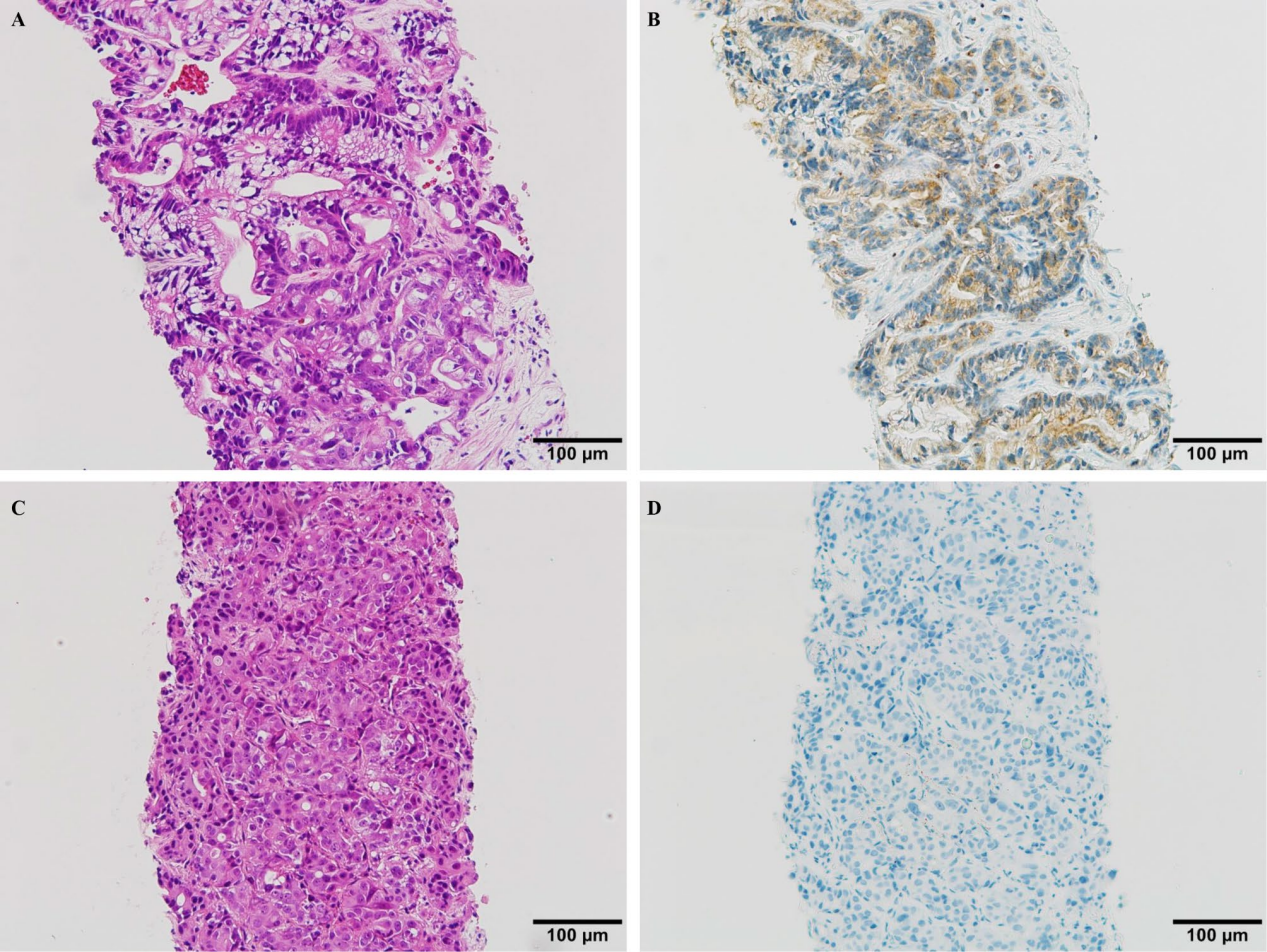
Representative immunohistochemical staining of Mcl-1 in specimens obtained using percutaneous ultrasound-guided liver tumor biopsy. Micrographs of pancreatic ductal carcinoma obtained using percutaneous ultrasound-guided liver tumor biopsy. (**A**) and (**B**): Well-differentiated adenocarcinoma (**A**: hematoxylin and eosin staining [HE], × 200) with more than 20% of the tumor cells in the cytoplasm showing immunoreactivity for Mcl-1 (**B**: immunohistochemical staining [IHC], × 200), which was considered positive. (**C**) and (**D**): Poorly differentiated adenocarcinoma (**C**; HE, × 200) with no cells showing immunoreactivity for Mcl-1 (**D**; IHC, × 200), which was considered a negative result.

The investigator, who was blinded to the clinical data, assessed the percentage of stained cells. Specimens with more than 20% stained cancer cells in the cytoplasm, excluding non-cancer cells such as fibroblasts or immune cells, were categorized as Mcl-1 positive, according to previously published protocols (Fig. 1)^22–24^.

### Statistical analysis

The χ^2^ test and Fisher’s exact test were used to compare categorical data, whereas the Wilcoxon rank sum text was used for the comparison of continuous and categorical variables. PFS and OS were estimated using Kaplan-Meier analysis, and the curves were compared using the log-rank test. Univariate and multivariate analyses were performed using the Cox regression method to evaluate the prognostic factors for PFS and OS. Variables with a P value < 0.20 in the univariate analysis were selected for inclusion into the multivariate analysis. Statistical analyses were performed using the JMP Pro 16 software (SAS Institute Inc., Cary, NC, USA). Statistical significance was set at *P* ≤ 0.05.

## Results

### Patient characteristics and the outcomes of GnP therapy

A total of 38 patients with MPC were included in this study. All the patients were histologically diagnosed as having pancreatic ductal adenocarcinoma (EUS-FNA, 25 patients; percutaneous liver tumor biopsy, 13 patients). EUS-FNA was performed with 19-gauge needle in 1 patient, 22-gauge needle in 16 patients, and 25-gauge needle in 6 patients (detailed information of the needles was not available for 2 patients). There were no cases of cyst arising tumors such as invasive intraductal papillary mucinous neoplasm in imaging and histological diagnosis. The clinical characteristics of the patients are summarized in Table 1. The median age of the patients was 65 years (range, 38–80 years). Eighteen patients (47.4%) were male. Thirty (78.9%) and eight (21.1%) patients had an Eastern Cooperative Oncology Group PS of 0 or 1 and 2, respectively. The median body mass index of the patients was 21.1 kg/m^2^ (range, 16.2–28.4 kg/m^2^). The primary tumor locations were the pancreas head in 12 patients (31.6%) and the pancreas body/tail in 26 patients (68.4%). Thirty-one patients (81.6%) had liver metastases, 8 patients (21.1%) patients had lung metastasis, and 15 patients (39.5%) had peritoneal metastasis. The median CEA, CA19-9, and albumin levels were 11 ng/mL (range, 1–338 ng/mL), 5484 U/mL (range, 2–100000 U/mL), and 3.6 g/dL (range, 2.4–4.5 g/dL), respectively. Fourteen patients (36.8%) had an NLR of less than 3. Biliary drainage was performed for eight patients (21.1%).

**Table 1:** Characteristics of the patients.

	Total (*n* = 38)	Mcl-1 positive (*n* = 23)	Mcl-1 negative (*n* = 15)	*P* value
Age, median (range), (years)	65 (38–80)	65 (38–80)	64 (53–76)	0.917
Sex, n (%)				
Female	20 (53.6)	11 (47.8)	9 (60.0)	0.463
Male	18 (47.4)	12 (52.2)	6 (40.0)	
ECOG PS, n (%)				
0	30 (78.9)	19 (82.6)	11 (73.3)	0.687*
1/2	8 (21.1)	4 (17.4)	4 (26.7)	
BMI, median (range), (kg/m^2^)	21.1 (16.2–28.4)	21.0 (16.2–28.2)	21.5 (17.5–28.4)	0.483
Tumor site, n (%)				
Pancreas body/tail	26 (68.4)	16 (69.6)	10 (66.7)	0.851
Pancreas head	12 (31.6)	7 (30.4)	5 (33.3)	
Metastatic sites, n (%)				
Liver	31 (81.6)	18 (78.3)	13 (86.7)	0.681*
Lung	8 (21.1)	6 (26.1)	2 (13.3)	0.440*
Peritoneum	15 (39.5)	8 (34.8)	7 (46.7)	0.514
CEA, median (range), (ng/mL)	11 (1-338)	13 (1-239)	7 (1-338)	0.332
CA19-9, median (range), (U/mL)	5484 (2-100000)	4077 (2-100000)	5693 (2-100000)	0.621
Albumin, median (range), (g/dL)	3.6 (2.4–4.5)	3.6 (3.0-4.5)	3.6 (2.4–4.1)	0.245
NLR, n (%)				
< 3	14 (36.8)	11 (47.8)	3 (20.0)	0.082
≧ 3	24 (63.2)	12 (52.2)	12 (80.0)	
Biliary drainage, n (%)				
No	30 (78.9)	20 (87.0)	10 (66.7)	0.223*
Yes	8 (21.1)	3 (13.0)	5 (33.3)	

Statistical significance was set at *p* < 0.05.

Chi-square test, *Fisher’s exact test.

ECOG PS, Eastern Cooperative Oncology Group performance status; BMI, body mass index; CEA, carcinoembryonic antigen; CA19-9, carbohydrate antigen 19 − 9; NLR, neutrophil-to-lymphocyte ratio.

Regarding the outcomes of GnP therapy, the response and disease control rates were 55.3% and 86.8%, respectively (Table 2). The median PFS was 5.7 months (range, 2.0–21.5 months), and the median OS was 11.2 months (range, 3.0–27.1 months). All patients died due to disease progression. While seven patients selected best supportive care after the discontinuation of GnP, 31 patients underwent subsequent chemotherapy (oral fluoropyrimidine S-1, 21 patients; modified FOLFIRINOX, 7 patients; clinical trial, 3 patients).

**Table 2:** Treatment outcomes for patients with MPC who received GnP as first-line chemotherapy.

	Total (*n* = 38)	Mcl-1 positive (*n* = 23)	Mcl-1 negative (*n* = 15)	*P* value
Best response, n				
CR	0	0	0	
PR	21	15	6	
SD	12	7	5	
PD	5	1	4	
NE	0	0	0	
Overall response rate, (%)	55.3	65.2	40.0	0.127
Disease control rate, (%)	86.8	95.7	73.3	0.046*

Statistical significance was set at *p* < 0.05.

Chi-square test, *Fisher’s exact test.

CR, complete response; PR, partial response; SD, stable disease; PD, progressive disease; NE, not evaluated.

### Association between Mcl-1 expression and outcomes of GnP therapy

**Fig. 2:**
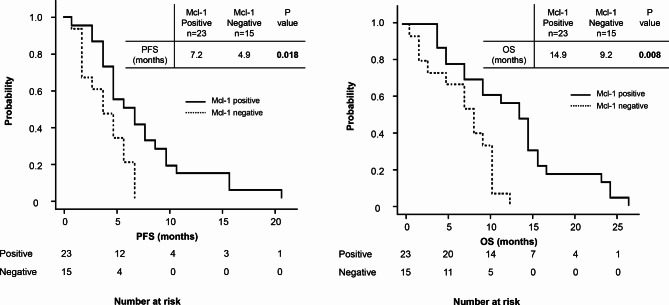
Comparison of progression-free survival (PFS) and overall survival (OS).

Of the 38 patients included in this study, 23 (60.5%) tested positive for Mcl-1 expression and 15 (39.5%) tested negative. Table 1 shows a comparison of patient characteristics between the Mcl-1 positive and negative groups. There were no significant differences in baseline characteristics between the two groups. The patients in the Mcl-1 positive group showed a significantly higher disease control rate than those in the Mcl-1 negative group (95.7% vs. 73.3%; *P* = 0.046, Table 2). In addition, the PFS and OS of the patients in the Mcl-1 positive group were significantly longer than those of the patients in the Mcl-1 negative group (PFS: 7.2 months [range, 2.0–21.5 months] vs. 4.9 months [range, 2.0–7.8 months], *P* = 0.018; OS: 14.9 months [range, 5.4–27.1 months] vs. 9.2 months [range, 3.0–13.3 months], *P* = 0.008; Fig. 2).

### Factors associated with PFS and OS

We analyzed the predictive factors associated with PFS in patients with MPC (Table 3). The univariate analysis showed that Mcl-1 expression was significantly associated with PFS (HR, 0.388; 95% CI, 0.182–0.822; *P* = 0.013). Multivariate analysis performed using three variables (male sex, NLR, and Mcl-1 expression) indicated that Mcl-1 expression was an independent predictor significantly associated with PFS (HR, 0.453; 95% CI, 0.210–0.975; *P* = 0.043).

**Table 3:** Univariate and multivariate analyses of factors associated with progression-free survival.

Factor	Univariate		Multivariate	
	HR (95%CI)	*P* value	HR (95%CI)	*P* value
Age				
≦ 65 years	1			
> 65 years	1.454 (0.722–2.926)	0.295		
Sex				
Female	1			
Male	0.584 (0.288–1.182)	0.135	0.648 (0.319–1.318)	0.231
ECOG PS				
0	1			
1 or 2	0.724 (0.313–1.673)	0.450		
BMI				
≦ 20 kg/m^2^	1			
> 20 kg/m^2^	1.282 (0.629–2.614)	0.494		
Tumor site				
Pancreas body/tail	1			
Pancreas head	0.791 (0.373–1.672)	0.539		
CEA				
≦ 5 ng/ml	1			
> 5 ng/ml	1.133 (0.564–2.275)	0.727		
CA19-9				
≦59×ULN	1			
>59× ULN	1.292 (0.656–2.543)	0.456		
Albumin				
≦3.5 g/dl	1			
>3.5 g/dl	1.171 (0.573–2.390)	0.664		
NLR				
≦ 3	1			
> 3	1.182 (0.913–3.606)	0.089	1.547 (0.766–3.120)	0.223
Drainage				
No	1			
Yes	0.878 (0.358–2.116)	0.760		
Mcl-1				
Negative	**1**			
Positive	**0.388 (0.182–0.822)**	**0.013**	**0.453 (0.210–0.975)**	**0.043**

Bold values indicate *P* < 0.05.

HR, hazard ratio; CI, confidence interval.

ECOG PS, Eastern Cooperative Oncology Group performance status; BMI, Body Mass Index; CEA, carcinoembryonic antigen; CA19-9, carbohydrate antigen 19 − 9; ULN, upper limit of normal; NLR, neutrophil-to-lymphocyte ratio.

We also evaluated the predictive factors associated with OS in patients with MPC (Table 4). The univariate analysis indicated that age (HR, 2.190; 95% CI, 1.028–4.667; *P* = 0.042) and Mcl-1 expression (HR, 0.276; 95% CI, 0.121–0.631; *P* = 0.002) were significantly associated with OS. Multivariate analysis performed using three variables (age, NLR, and Mcl-1 expression) revealed that Mcl-1 expression was an independent predictor significantly associated with OS (HR, 0.321; 95% CI, 0.136–0.759; *P* = 0.010).

**Table 4:** Univariate and multivariate analyses of factors associated with overall survival.

Factor	Univariate		Multivariate	
	HR (95%CI)	*P* value	HR (95%CI)	*P* value
Age				
≦ 5 years	1			
> 65 years	**2.191 (1.028–4.667)**	**0.042**	1.841 (0.837–4.047)	0.129
Sex				
Female	1			
Male	0.829 (0.422–1.628)	0.585		
ECOG PS				
0	1			
1 or 2	0.705 (0.299–1.661)	0.424		
BMI				
≦ 20 kg/m^2^	1			
> 20 kg/m^2^	1.189 (0.595–2.377)	0.625		
Tumor site				
Pancreas body/tail	1			
Pancreas head	0.868 (0.421–1.787)	0.701		
CEA				
≦ 5 ng/ml	1			
> 5 ng/ml	0.974 (0.499-1.900)	0.938		
CA19-9				
≦ 59×ULN	1			
> 59× ULN	1.005 (0.522–1.936)	0.988		
Albumin				
≦ 3.5 g/dl	1			
> 3.5 g/dl	0.825 (0.418–1.623)	0.577		
NLR				
≦ 3	1			
> 3	1.854 (0.946–3.635)	0.072	1.5330 (0.645–2.743)	0.440
Drainage				
No	1			
Yes	1.220 (0.554–2.681)	0.621		
Mcl-1				
Negative	1			
Positive	**0.276 (0.121–0.631)**	**0.002**	**0.321 (0.136–0.759**)	**0.010**

Bold values indicate *P* < 0.05.

HR, hazard ratio; CI, confidence interval.

ECOG PS, Eastern Cooperative Oncology Group performance status; BMI, Body Mass Index; CEA, carcinoembryonic antigen; CA19-9, carbohydrate antigen 19 − 9; ULN, upper limit of normal; NLR, neutrophil-to-lymphocyte ratio.

## Discussion

Expression of Mcl-1, an anti-apoptotic protein, is elevated in PC. However, the clinical significance of Mcl-1 expression in PC and its association with therapeutic response are unclear^25,26^. In the present study, the PFS and OS of the patients in the Mcl-1 positive group were significantly better than those of patients in the Mcl-1 negative group. Furthermore, multivariate analysis showed that Mcl-1 expression was an independent predictive marker for favorable PFS and OS. While it has been reported that PC with elevated Mcl-1 expression has a poor prognosis, Mcl-1 inhibitors induce apoptosis more strongly in tumors with elevated Mcl-1^19,27^. Gemcitabine has been reported to downregulate Mcl-1 expression and induce apoptosis in PC cells^28,29^. It is also reported that Mcl-1 is an important factor in determining the response to paclitaxel treatment, and the downregulation of Mcl-1 restores sensitivity to paclitaxel^30,31^. Together with these previous reports, favorable treatment responses of GnP in PC with elevated Mcl-1 expression may be possibly explained by downregulation caused by gemcitabine and subsequent restoration of sensitivity to paclitaxel.

GnP and fluorouracil-containing chemotherapeutic regimens (modified FOLFIRINOX and NALIRIFOX) are important treatment options for patients with MPC^32,33^. However, there are few clinical indicators of whether GnP or fluorouracil-containing regimens should be selected for chemotherapy. In the present study, patients with elevated Mcl-1 expression showed better treatment outcomes than those without elevated Mcl-1 expression. This finding suggests that Mcl-1 expression in tissue samples obtained by biopsy could be a predictive marker of the therapeutic effects of GnP. Studies conducted using comprehensive genomic profiling (CGP) tests have revealed that approximately 20% of patients with pancreatic ductal adenocarcinoma show mutations in homologous recombination genes, including *BRCA1/2* and *PALB2*^34,35^. Moreover, patients with homologous recombination gene-mutated PC show favorable PFS and OS with platinum-containing chemotherapeutic regimens^35,36^. Mutations in homologous recombination genes are promising predictive markers of response to chemotherapy and can guide the selection of appropriate regimens. However, given that it takes a long time to obtain the results of a CGP test, it is difficult to factor in the presence of these mutations in the selection of chemotherapeutic regimens before initiating chemotherapy^37–39^.

The results of the present study demonstrated that Mcl-1 expression in specimens obtained using EUS-FNA or percutaneous liver tumor biopsy could predict the efficacy of chemotherapy. EUS-FNA is the primary method used for the pathological diagnosis of pancreatic tumor^40–42^. With the recent improvements in puncture needles, histological samples have also been used for CGP tests^43–45^. Percutaneous liver tumor biopsy also plays an important role in pathological diagnosis and tissue acquisition for genetic testing^46,47^. As immunohistological examinations are simple and rapid, Mcl-1 expression in specimens can be analyzed prior to the initiation of chemotherapy. Thus, tissue samples obtained by EUS-FNA or percutaneous liver tumor biopsy may contribute not only to the diagnosis of PC but also to the selection of chemotherapeutic regimens as well.

This study has some limitations. First, this was a single-center retrospective study that examined cases meeting specific criteria, and it is unclear whether the findings can be generalized to patients with advanced PC who are undergoing GnP therapy in general. Second, we did not examine patients with MPC who received modified FOLFIRINOX as a first-line chemotherapeutic regimen, primarily because the number of patients who received the therapy was small. Further studies are needed to clarify the mechanism by which Mcl-1 influences the therapeutic effects of GnP.

In conclusion, this study demonstrated that Mcl-1 may be a predictive marker for the therapeutic effect of GnP in patients with MPC. This suggests that immunohistological examination of Mcl-1 expression before initiation of treatment could facilitate the selection of appropriate chemotherapeutic regimens.

## Data Availability

Data supporting the findings of this study are available upon reasonable request from the corresponding author.
